# How annual course of photoperiod shapes seasonal behavior of diploid and triploid oysters, *Crassostrea gigas*

**DOI:** 10.1371/journal.pone.0185918

**Published:** 2017-10-11

**Authors:** Laura Payton, Mohamedou Sow, Jean-Charles Massabuau, Pierre Ciret, Damien Tran

**Affiliations:** 1 University of Bordeaux, EPOC, UMR 5805, Arcachon, France; 2 CNRS, EPOC, UMR 5805, Arcachon, France; University of Ferrara, ITALY

## Abstract

In this work, we study if ploidy (*i*.*e*. number of copies of chromosomes) in the oyster *Crassostrea gigas* may introduce differences in behavior and in its synchronization by the annual photoperiod. To answer to the question about the effect of the seasonal course of the photoperiod on the behavior of *C*. *gigas* according to its ploidy, we quantified valve activity by HFNI valvometry *in situ* for 1 year in both diploid and triploid oysters. Chronobiological analyses of daily, tidal and lunar rhythms were performed according the annual change of the photoperiod. In parallel, growth and gametogenesis status were measured and spawning events were detected by valvometry. The results showed that triploids had reduced gametogenesis, without spawning events, and approximately three times more growth than diploids. These differences in physiological efforts could explain the result that photoperiod (daylength and/or direction of daylength) differentially drives and modulates seasonal behavior of diploid and triploid oysters. Most differences were observed during long days (spring and summer), where triploids showed longer valve opening duration but lower opening amplitude, stronger daily rhythm and weaker tidal rhythm. During this period, diploids did major gametogenesis and spawning whereas triploids did maximal growth. Differences were also observed in terms of moonlight rhythmicity and neap-spring tidal cycle rhythmicity. We suggest that the seasonal change of photoperiod differentially synchronizes oyster behavior and biological rhythms according to physiological needs based on ploidy.

## Introduction

Valve opening activity in many bivalves species is closely related to physiological processes such as respiration, nutrition, and reproduction, which are modulated by environmental parameters [1,2]. As a marine organism, valve behavior of the oyster *Crassostrea gigas* is driven in its biotope by the multiple environmental cycles linked to periodical recurrence of sun-earth-moon orbital positions [3]. Specifically, valve opening duration of permanently immersed diploid oysters follows a strong tidal cycle, modulated by synodic moon cycles (neap-spring tides) and anomalistic moon cycles (based on moon—earth distance) [3]. However, until now no moonlight effect has been shown in diploid oysters. Additionally, a circadian clock was demonstrated in *C*. *gigas*, synchronizing with the daily solar cycle to run at 24h [3–5]. This circadian clock allows the anticipation of light / dark alternation, and an internal temporal organization of biological processes [5,6]. In situ, the daily rhythm of subtidal oyster behavior was less expressed than the circatidal rhythm [3].

The year, a far-reaching environmental cycle of about 365 days caused by the earth’s revolution around the sun, is reflected by an entirely predictable annual change of photoperiod at any given latitude and leads to seasonal changes in the environment. Temporal coordination with annual cycles is crucial for the maintenance of fitness in organisms, as seasonality commonly implies alternations in the availability of conditions that are conducive to growth and gametogenesis [7]. It is now well documented that some species exhibit an endogenous circannual rhythm, synchronized by photoperiod, in which the circadian clock should provide a reference clock for the reading of calendar information [8–10]. Seasonal cycles are observed in the Pacific oyster, *C*. *gigas*, especially the widely described reproductive cycle [11–14]. Others biological processes such as immune parameters, catecholamine levels, growth, metabolism or biochemical content also show seasonal variations [13–19]. However, the importance of seasonal cycles in shaping valve behavior is globally unknown. A previous study highlighted a dualism in the circadian rhythm of valve opening duration, characterized by a switch from nocturnal valve activity in autumn and winter to diurnal activity in spring and summer [4]. Switches happened in the field and laboratory, where animals were disconnected from field conditions, suggesting an internal origin for this annual rhythm [4]. Thus, seasonal oscillations would come from oysters themselves, which would be sensitive to photoperiodic information as zeitgeber to synchronize a putative endogenous circannual rhythm, leading to synchronized seasonal biology.

The Pacific oyster *Crassostrea gigas* has been widely introduced and dominates bivalve production for human consumptions in many regions [20–22]. A common method of increasing commercialization and production of this marine mollusk is induced triploidy, a physiological status in which organisms have three sets of chromosomes as opposed to the normal two sets in diploids. Triploidy mainly confers reduced gonadic development, hence it facilitates commercialization over the summer, and faster growth [23–28]. The molecular mechanisms by which polyploidy contributes to novel phenotype variation are not well understood. The augmented genetic material in triploids can lead to increased heterozygoty, as well as potential genome rearrangements or modifications of repeated elements, and thus possibly novel forms of regulation of gene expression and modification of transcription [29–32]. In *C*. *gigas*, triploidy led to modification in immune parameters, in lipid content, or in toxin bioaccumulation during harmful algae bloom exposure [17,23,26,33]. Moreover, triploids appeared less sensitive to environmental cues concerning seasonality of immune parameters [17]. Despite their wide use, however, triploid oyster behavior in the ecosystem is not known and the impact of triploid status on valve behavior and its synchronization with environmental factors remains to be elucidated.

In the present study, the first aim was to further understand seasonal valve activity behavior of *C*. *gigas* including how it is shaped by photoperiodism and lunar cycles. Then, we aimed to analyze if triploid status could modify oyster behavior and its synchronization with environmental cycles. For that, we took advantage of the high-frequency noninvasive valvometry technique that allowed one full year of continuous recording of *in situ* animal valve movements without any human intervention [34].

We analyzed the seasonal course of photoperiod in the environment on several behavioral parameters including: valve opening duration and amplitude; daily, tidal and lunar month rhythmicity; growth index; and spawning events in the Bay of Arcachon (France) in subtidal conditions.

## Materials and methods

### Animals

The study was carried out on 94 diploid and 94 triploid Pacific oysters *Crassostrea gigas* comparable in shell length (65.8 ± 2.0 mm, 24 months-old and 66.6 ± 1.4 mm, 18 months-old, respectively), both purchased in February 2014 from oyster farmers in front of the Marine Station at the Bay of Arcachon (S.W. France). Oysters *C*. *gigas* are not endangered or protected species. Diploid oysters were collected by the farmers ("Port du Rocher", La Teste de Buch, Arcachon bay, France, Lat. 44°38'N, Long. 1°7'O) from natural settlements and triploids were purchased by the farmers (« Les 3B », Gujan Mestras, Arcachon bay, France, Lat. 44°64’N, Long. 1°08’O) from a commercial hatchery (“Grainocean”, Saint-Martin-de-Ré, France, Lat. 46°2’N, Long. 1°35’O) which used many diploids (≈ 200 females) and quadriploids (10 males) parents, and thus limited parenting effect. Both diploids and triploids oysters were cultivated in “Grand Banc”, Bay of Arcachon. The oysters were positioned in the field site (Eyrac Pier, Bay of Arcachon, Lat. 44°66’N, Long. 1°16’O, no specific permissions were required for this location and this scientific activity, done with native oysters) on February 14, 2014 in the proximity of wild oysters. They were inside a permanently immersed oyster bag (1 × 0.5 m) fixed on an oyster table for valvometry measurement (mix of diploids and triploids oysters); two others oyster bags (one for morphogenesis monitoring and the other one for gametogenesis monitoring) both with mix of diploids and triploids oysters, were suspended from a chain at ≈ 0.4 m above the seafloor, near the bag of valvometry. The oysters were always in subtidal conditions, at a minimum water depth of ≈ 1 m. The tides were semidiurnal, *i*.*e*. 2 tides per day, with an amplitude maximal ± 2.4 m and a maximal tidal flow at mid-tide reaching ≈ 0.5–0.6 m.s^-1^ [35]. The astronomical data related to the sun, earth, and moon positions were provided from the site www.imcce.fr.

All experiments presented in this paper complied with the laws in effect in France, where they were performed, and they conformed to international ethical standards.

### Morphogenesis and gametogenesis monitoring

Sixteen diploid oysters and 16 triploid oysters were followed for morphogenesis monitoring throughout the 1-yr study. Shell length, shell width, and total fresh body weight were measured prior to positioning in the field site, and 7 times (14/02/14, 17/03/14, 12/05/14, 16/07/14, 11/09/14, 04/11/14, and 08/01/15). Additionally, oyster gametogenesis was estimated by gonad observation after sacrifice for 10 diploid and 10 triploid oysters at each time. Gametogenesis status was according to the scale of Marteil [36]. This scale ranges from 0 to 3 where level 0 corresponds to an absence of gonads by visual inspection and level 3 to the most advanced level of gonadal development just before spawning. An additional observation date was added after the first spawning event, 29 July 2014.

### Photoperiod

**Fig 1:**
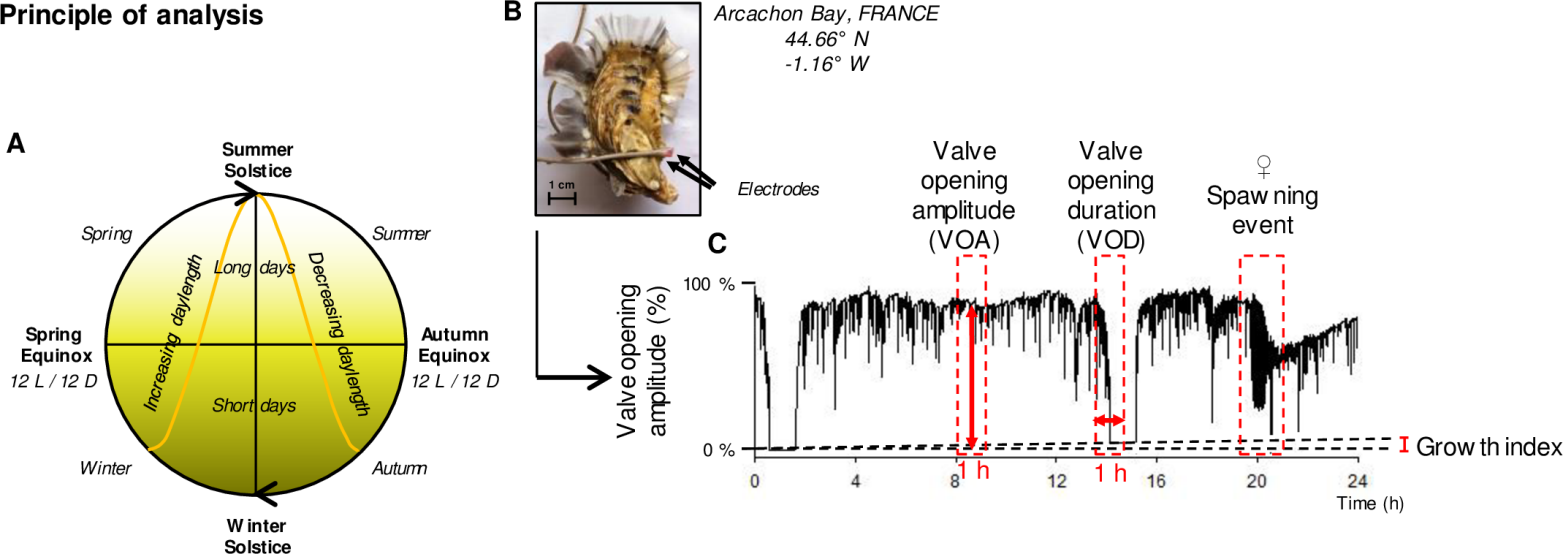
Principle of analysis. **(**A) Schematic representation of an annual cycle where seasons are characterized by two photoperiod parameters: daylength (long or short days) and direction of daylength (increasing or decreasing daylength). L for light, D for dark. (B) Example of light electrodes of high frequency non-invasive valvometry glued to one of the triploid oysters deployed in Arcachon bay (France), for the one-year recording in the field. The black arrows indicate the position of the electrodes glued on opposite edges of the shells. Only the electrode glued on the superior valve is apparent in this picture. (C) Typical records of valve opening behavior of *Crassostrea gigas* during a one-day period (24 h). Four parameters were used in this study: hourly valve opening amplitude (VOA), hourly valve opening duration (VOD), spawning events and growth index corresponding to evolution of minimum valve opening over time (schematized in the one-day period in this figure).

Photoperiod is the predictable annual change of daylength throughout a year (Fig 1A). The four seasons caused by the earth’s revolution around the sun at any given latitude are delimited by the predictable events of equinoxes and solstices. The spring and autumn equinoxes, when day and night are the same length (12 L / 12 D), marks the beginning of spring and autumn, the 20^th^ of March 2014 and the 21^st^ of September 2014, respectively. The longest day duration is reached at the summer solstice and marks the beginning of summer, the 21^st^ of June 2014. The shortest daylength is reached at the winter solstice and marks the beginning of winter, the 20^th^ of December 2014. Thus, for each season, photoperiod is characterized by two parameters. The first one is the daylength, described in this study as “long” when day is longer than night (*i*.*e*. photophase > 12 h, in spring and summer) and “short” when day is shorter than night (i.e. photophase < 12 h, in autumn and winter). The second one is the direction of daylength: increasing (winter and spring) or decreasing (summer and autumn) photoperiod.

### High-frequency noninvasive (HFNI) valvometry

The *in situ* valve movement behavior of the bivalves was recorded using a high-frequency noninvasive (HFNI) valvometer technology, described by [3,37]. Briefly, a pair of lightweight electrodes designed to minimize disturbance to bivalve behavior, were glued on each half shell (Fig 1B). Between the electrodes, an electromagnetic current was generated, which allowed measurement of the amount of valve opening and closing. The signal was recorded using a custom acquisition card every 0.1 s. Because 16 oysters were monitored, each oyster was measured every 1.6 s and the data were automatically transmitted daily to a data processing center in the Arcachon Marine Biological Station (France) using cellular and Internet networks and were reported daily on the Molluscan Eye website (https://molluscan-eye.epoc.u-bordeaux.fr/).

The present study analyzed data from 20 March 2014 (Spring Equinox 2014) to 19 March 2015 (Spring Equinox 2015), on 8 diploid and 8 triploid oysters. During this 1-y analysis, two periods of missing data occurred: from 21 to 31 July 2014 (11 days) and from 6 to 14 October 2014 (9 days), due to electrical issues.

### HFNI valvometry data treatment

#### Valve opening amplitude and valve opening duration

Each day of the year, individual valve opening amplitudes of each oyster were reported in percentage (Fig 1C). Then, on an hr-per-hr and individual basis, two parameters were reported: the amount of time the valves of each oyster were opened (below 5% of valve opening amplitude, the oyster is considered closed), called valve opening duration (VOD, %); and the amplitude of the opening (relatively to maximum opening), called valve opening amplitude (VOA, %) (Fig 1C). Individual hourly VOD and VOA were averaged based on ploidy status (diploid *vs* triploid). A VOD of 100% meant that the valves of all animals were at least partially open during the 1 hour period and 0% that no animals had open valves. A VOA of 100% meant that the valves of all animals were maximally opened for the 1 hour period and 0% that no animals had open valves. Daily VOD and VOA values were the result of the average of the 24 hours of each day. A chronobiological analysis was performed every 15 days, according to the moon Neap Spring Tidal cycle (*i*.*e*. new moon to full moon or full moon to new moon), dividing the 365 days of data into 25 parts.

#### Growth index

In bivalve mollusks, calcification takes place in the mantle cavity, over the shell’s internal surface. As a result, the consequence of daily growth was an increase in the minimal distance between electrodes when valves were closed, i.e. the minimum valve opening amplitude (min) (schematized in a one day record in Fig 1C). Thus, growth index was calculated individually by isolating daily values for minimum valve opening amplitude, and then averaged according to ploidy status (diploid *vs* triploid). For each 15-day analysis, mean growth index was set to 0 on day 1. The growth rate index was calculated as follow: Δ min (day x_[__1__–__15__]_−day 1) of distance between electrodes / Δ duration (day x_[__1__–__15__]_−day 1).

#### Spawning events

Spawning events of female oysters have already been characterized by HFNI valvometry [35,38]. Briefly, female spawning corresponds to a typical pattern of valve movement comprising a burst of rhythmic valve closures, which males do not show [39,40]. The beginning of a spawning event was considered as the beginning of the first adductor muscle contraction in a burst.

### Chronobiological analysis

#### Actograms

Double-plotted actograms were produced with Chronos-Fit 1.05, starting at 20:00 h. Each line represented 2 days (365 lines in our case). By convention, each 2-day period was first represented from 24 to 48 h, and then repeated on the next line from 0 to 24 h. The black and white sections of each line represent hourly behavioral values above and below the mean behavior of the day, respectively.

#### Statistical chronobiological analyses

Chronobiological analyses were performed using the software Time Series Analysis Seriel Cosinor 6.3. Several steps were required to validate a significant rhythm [41–43].

#### Quality of the data set

The absence of random distribution in the data set was controlled using an autocorrelation diagram, and the absence of a stationary phenomenon by a partial autocorrelation function calculation [44]. These checks indicated a real biological or physical phenomenon.

#### Search for periodicity

The Lomb and Scargle periodogram was used to determine the period in the equispaced data [45]. To be accepted, a period needed to be significant with the Lomb and Scargle periodogram (*p* = 0.95).

#### Modeling and statistical validation

Rhythmicity was modeled with the Cosinor model, which uses a cosine function calculated by regression [46,47]. For a given period, the model is written as: *Y* (*t*) = Acos (*πt*/*τ* + *ϕ*) + *M* + *ε* (t), where A is the amplitude, *ϕ* the acrophase, *τ* the period, *M* the mesor and *ε* the relative error [41,42]. Two key tests validated the calculated model and the existence of a rhythm: the elliptic test [46] had to be rejected and the probability for the null amplitude hypothesis had to be <0.05. A chronobiometric parameter was calculated: the percent rhythm (PR, %), which is the percentage of cyclic behavior explained by the model. Rhythms with different periods might be superimposed on a single dataset. For 15-day data, we analyzed the first two periods expressed by the oysters: tidal rhythm (τ = 12.4 h) and daily rhythm (τ = 24 h). For 1-yr data, moonlight effect (τ = 29.53 d) and Neap-spring tidal cycle (τ = 14.76 d) of the lunar synodic cycle was analyzed.

### Statistical analyses

Results were expressed as mean ± 1 SE. Three-way analyses of variance (ANOVA) were performed after checking assumptions (normality of data and homoscedasticity of the error term). In case of significant differences, Student-Newman-Keuls method was applied for all pairwise multiple comparisons. Kruskal-Wallis one-way analysis of variance on ranks and Mann-Whitney rank sum test were applied for multiple comparisons and for two group comparisons respectively when data failed normality tests. Paired t-tests were used after checking assumptions (normality of data) for seasonal comparison of diploid and triploid rhythms. For all statistical results, a probability of *p* < 0.05 was considered significant. Statistical analyses were performed using Sigma Plot software (Version 13.0; Systat Software, USA).

## Results

### Valve opening duration and valve opening amplitude

**Fig 2:**
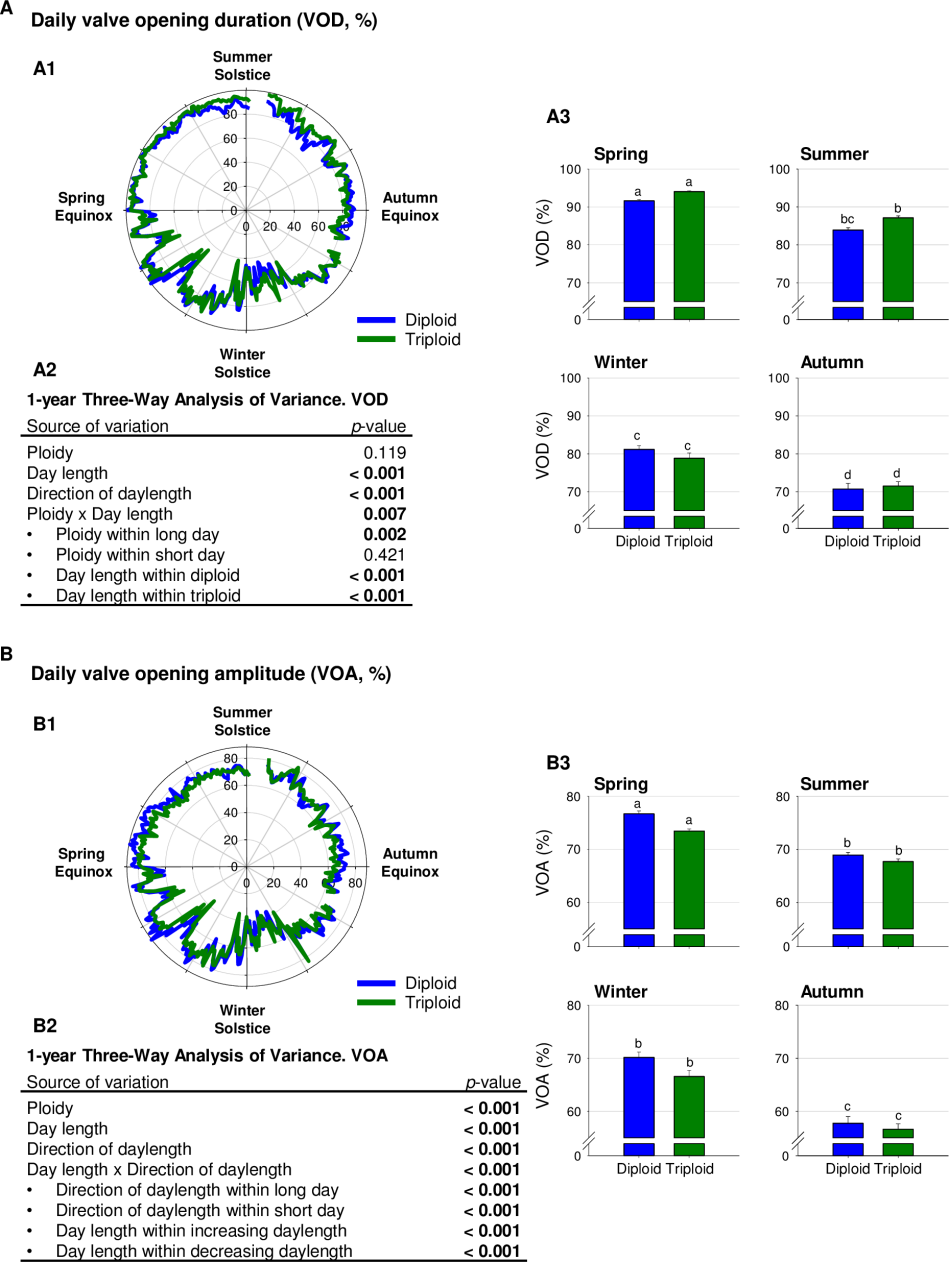
Valve opening duration (VOD) and amplitude (VOA). **(**A) Mean daily valve opening duration (VOD, %) of diploid (blue, n = 8) and triploid (green, n = 8) oysters during the 1-year analyses. (A1) Polar plot representation of mean daily VOD. (A2) Three-way analysis of variance of VOD using ploidy (diploid or triploid), daylength (long or short days) and direction of daylength (increasing or decreasing daylength). (A3) Seasonal histograms of VOD. Letters indicate significant differences (one-way analysis of variance, *p* < 0.05). (B) Mean daily valve opening amplitude (VOA, %) of diploid (blue, n = 8) and triploid (green, n = 8) oysters during the 1-year analyses. (B1) Polar plot representation of VOA. (B2) Three-way analysis of variance of VOA using ploidy (diploid or triploid), daylength (long or short days) and direction of daylength (increasing or decreasing daylength). B3. Seasonal histograms of VOA. Letters indicate significant differences (one-way analysis of variance, *p* < 0.05).

Fig 2A1 shows the mean daily valve opening duration (VOD, %) of diploid and triploid oysters from spring equinox 2014 to spring equinox 2015. At the annual scale, a three-way ANOVA showed no global ploidy effect on daily VOD (Fig 2A2). However, there was a significant ploidy effect during long days (spring and summer), which disappeared during short days (autumn and winter). Triploid oysters were open for a longer daily duration than diploid during long days. Furthermore, the three-way ANOVA interactions showed that both daylength (short days *vs* long days) and direction of daylength (increasing *vs* decreasing photoperiod) significantly influenced daily VOD in all oysters (Fig 2A2). Valve opening duration was longer during long days and with increasing photoperiod. At the seasonal scale, a one-way ANOVA (Fig 2A3) showed that oysters had a maximum daily VOD in spring (91.6 ± 0.4% in diploid and 94.0 ± 0.2% in triploid) and a minimum daily VOD in autumn (70.7 ± 1.5% in diploid and 71.5 ± 1.1% in triploid). In diploids, there were significant differences between seasons except summer (83.9 ± 0.6%) *vs* winter VOD (81.1 ± 1.0%) while in triploids (87.1 ± 0.5% in summer, 78.8 ± 1.4% in winter), all seasons were significantly different from each other.

Fig 2B1 shows the mean daily valve opening amplitude (VOA, %) at the annual scale.

The three-way ANOVA (Fig 2B2) revealed a significant impact of ploidy on the mean daily VOA. Triploid oysters presented a significant lower VOA than diploid oysters throughout the year. The three-way ANOVA also revealed a significant effect of both daylength and direction of daylength on VOA without ploidy distinction (Fig 2B2). Moreover, significant interactions between these two factors in all oysters included direction of photoperiod within long (spring VOA differed from summer) and short days (winter VOA differed from autumn), as well as daylength within increasing (winter VOA differed from spring) and decreasing days (summer VOA differed from autumn). At the seasonal level, a one-way ANOVA (Fig 2B3) confirmed maximum daily VOA in spring (76.7 ± 0.6% in diploid and 73.5 ± 0.4% in triploid), minimum in autumn (57.7 ± 1.3% in diploids and 56.6 ± 1.0% in triploids), while VOA in summer (68.9 ± 0.5% in diploid and 67.7 ± 0.5% in triploid) and winter (70.2 ± 1.1% in diploid and 66.6 ± 1.2% in triploid) were intermediate in both diploid and triploid oysters.

### Tidal and daily rhythms

**Fig 3:**
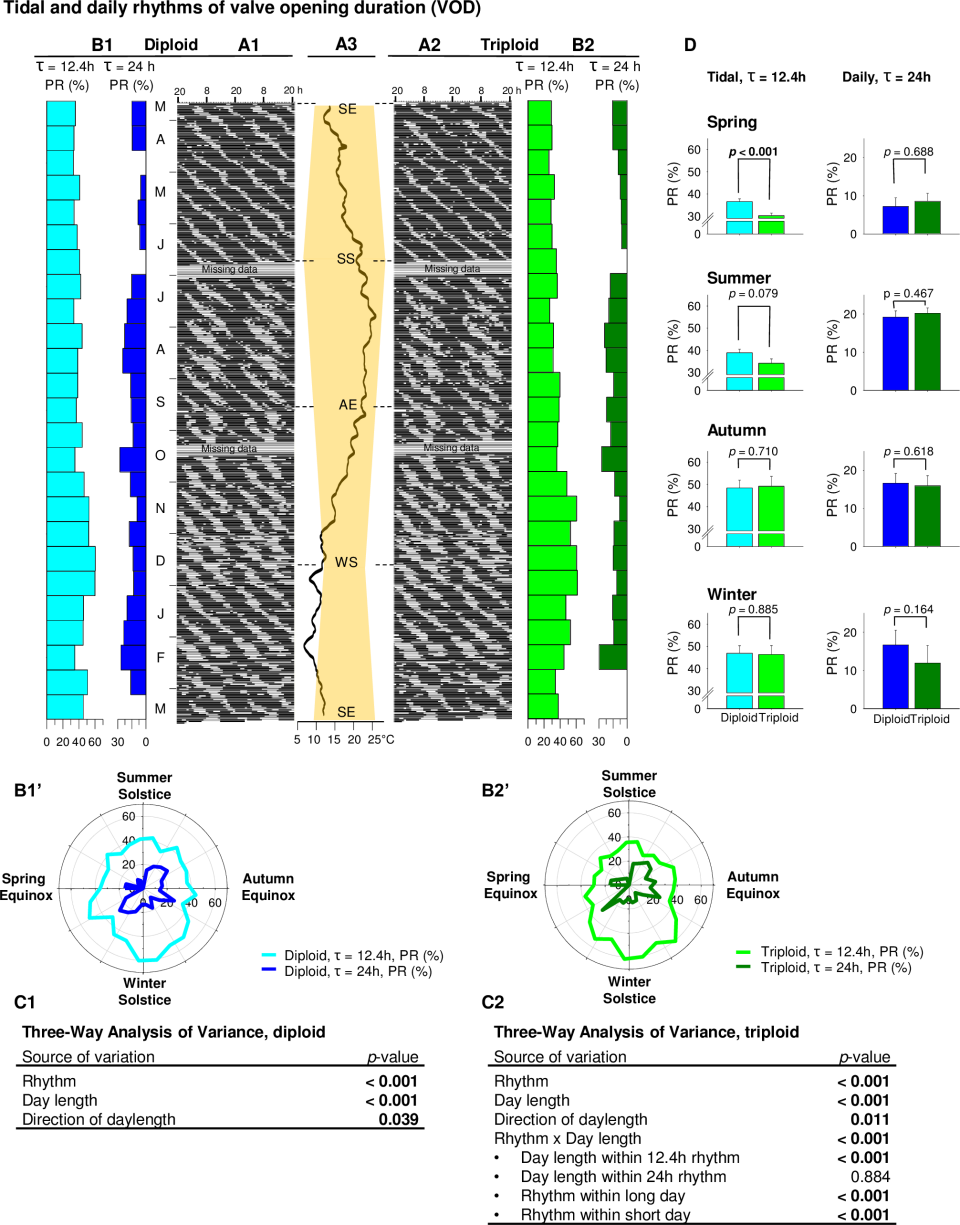
Tidal and daily rhythms of valve opening duration (VOD). **(**A) Actograms show the 1-year pattern of VOD in diploids (n = 8) (A1) and triploids (n = 8) (A2) Letters indicate either months (form March 2014 to March 2015) or equinoxes (SE: summer equinox, AE: autumn equinox) and solstices (SS: summer solstice, WS: winter solstice). (B) Percent rhythms (PR, %) of tidal (light blue for diploids or light green for triploids) and daily (dark blue for diploids or dark green for triploids) rhythms for each 15-day part of the 1-year analysis of VOD in diploids (B1) and triploids (B2). Zero values mean no significant rhythms. Same results presented as polar plots for diploid (B1’) and triploid (B2’) oysters. (C) Three-way analysis of variance on percent rhythms data using rhythm (tidal or daily), daylength (long or short days) and direction of daylength (increasing or decreasing daylength) in diploid (C1) and triploid (C2) oysters. (D) Seasonal histograms of tidal and daily percent rhythms data in diploid (blue) and triploid (green) oysters. *P*-values indicate differences between diploid and triploid oysters in a given season for tidal and/or daily rhythms.

Actograms showed the main pattern of diploid (Fig 3A1) and triploid (Fig 3A2) VOD from spring equinox 2014 to spring equinox 2015, with in parallel the annual photoperiod and water temperature measured near the oysters (Fig 3A3). The VOD distribution was clearly not random, but followed the semi-diurnal tidal cycle (*i*.*e*. two tidal cycles 12.4 h / day), with two main periods of lower opening duration (white sections) per day, synchronized to low tide slacks. Also, a diurnal rhythm was observed, with one main period of lower opening duration per day. Chronobiological analyses of 15-day periods revealed the percentage of cyclic behavior (percent rhythm (PR), %) explained by tidal (τ = 12.4 h) and daily rhythms (τ = 24 h), when significant rhythms were determined (Fig 3B1 and 3B2), presented in a circular time scale (Fig 3B1’ and 3B2’). Null PRs corresponded to non-significant rhythms. Three-way ANOVA showed a significant difference between tidal and daily PRs in both ploidies: tidal VOD behavior was stronger than circadian VOD behavior throughout the year (Fig 3C1 and 3C2). In diploid oysters, tidal and daily PRs were significantly affected by both daylength and direction of photoperiod. VOD rhythms (tidal and daily) were stronger during short days (autumn and winter) than long (spring and summer), and during decreasing photoperiod (summer and autumn) than increasing (winter and spring) (Fig 3C1). In triploid oysters, daylength had a significant effect on the VOD tidal rhythm (stronger during short days than long days) but not on the daily one, while the direction of photoperiod affected both rhythms (stronger during decreasing days than increasing) (Fig 3C2). At the seasonal level, the VOD tidal rhythm in diploids and triploids was significantly different only in the spring (Fig 3D).

**Fig 4:**
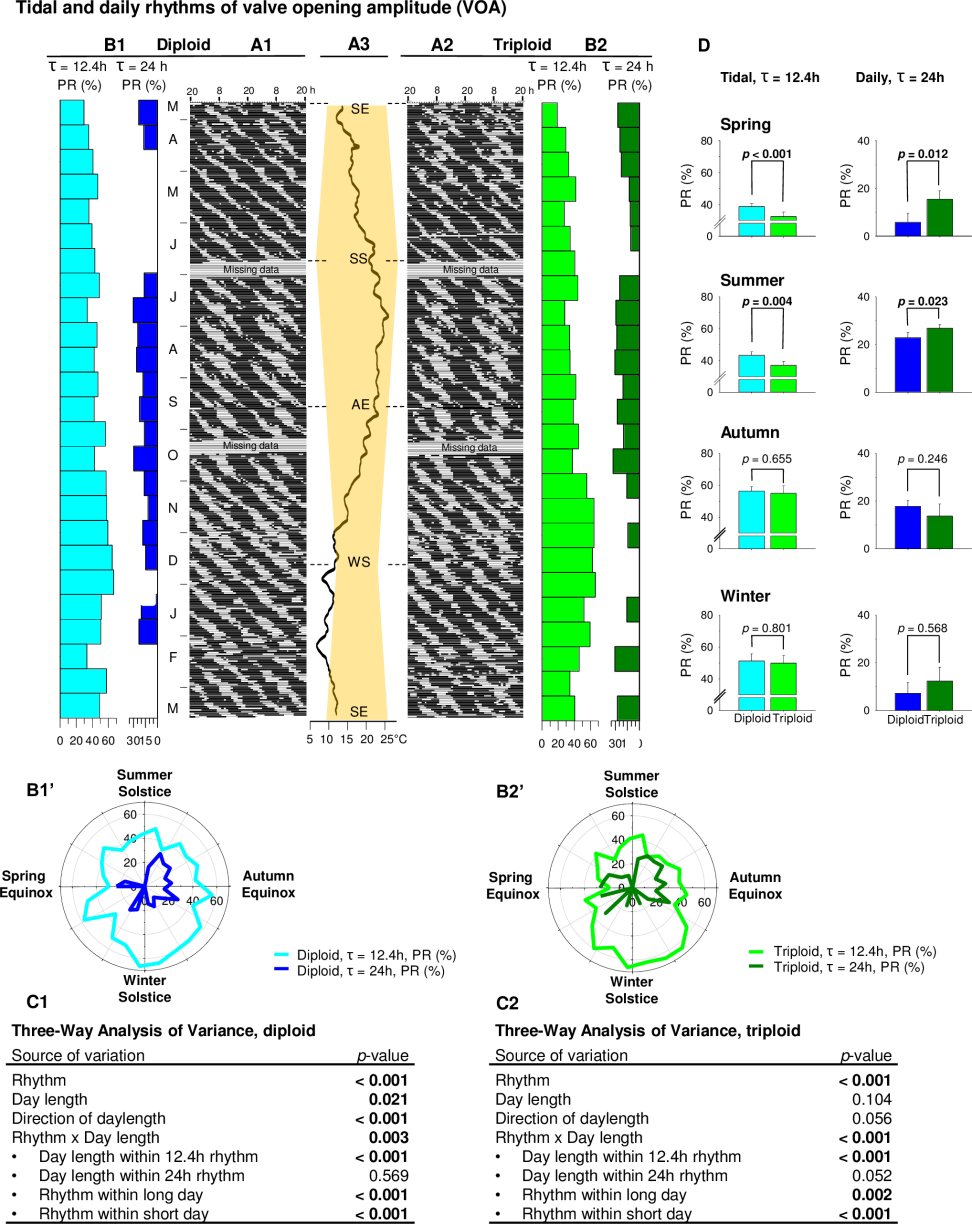
Tidal and circadian rhythms of valve opening amplitude (VOA). **(**A) Actograms show the 1-year pattern of VOA in diploids (n = 8) (A1) and triploids (n = 8) (A2). Letters indicate either months (form March 2014 to March 2015) or equinoxes (SE: summer equinox, AE: autumn equinox) and solstices (SS: summer solstice, WS: winter solstice). (B) Percent rhythms (PR, %) of tidal (light blue for diploids or light green for triploids) and daily (dark blue for diploids or dark green for triploids) rhythms for each 15-day part of the 1-year analysis of VOA in diploid (B1) and triploid (B2) oysters. Zero values mean no significant rhythms. Same results presented as polar plots for diploid (B1’) and triploid (B2’) oysters. (C) Three-way analysis of variance on percent rhythms using rhythm (tidal or daily), daylength (long or short days) and direction of daylength (increasing or decreasing daylength) data in diploid (C1) and triploid (C2) oysters. (D) Seasonal histograms of tidal and daily percent rhythms data in diploid (blue) and triploid (green) oysters. *P*-values indicate differences between diploid and triploid oysters in a given season for tidal and/or daily rhythms.

Fig 4 shows the same results as Fig 3, mean valve opening amplitude (VOA) behavior, but here three-way ANOVA on diploid and triploid oysters independently showed a significant difference between tidal and daily rhythms in both ploidies: tidal VOA behavior was stronger than daily throughout the year (Fig 4C1 and 4C2). In diploid oysters, daylength affected only the VOA tidal rhythm and not the daily one: tidal rhythm was stronger during short days than during long days (Fig 4C1). However, both tidal and daily VOA rhythms were affected by the direction of daylength: they were stronger during decreasing photoperiod (Fig 4C1). Similarly, in triploid oysters, only VOA tidal rhythm was impacted by daylength, with stronger tidal PRs during short days (Fig 4C2). However, in triploid oysters, neither VOA tidal nor daily rhythms were significantly affected by direction of daylength (Fig 4C2). There were no significant interactions between a rhythm and the direction of daylength. At the seasonal level, shown Fig 4D, a significant ploidy effect was revealed in both tidal and daily rhythms during the spring and summer: tidal rhythm was stronger in diploids whereas daily rhythm was stronger in triploids.

### Circalunar rhythms

**Fig 5:**
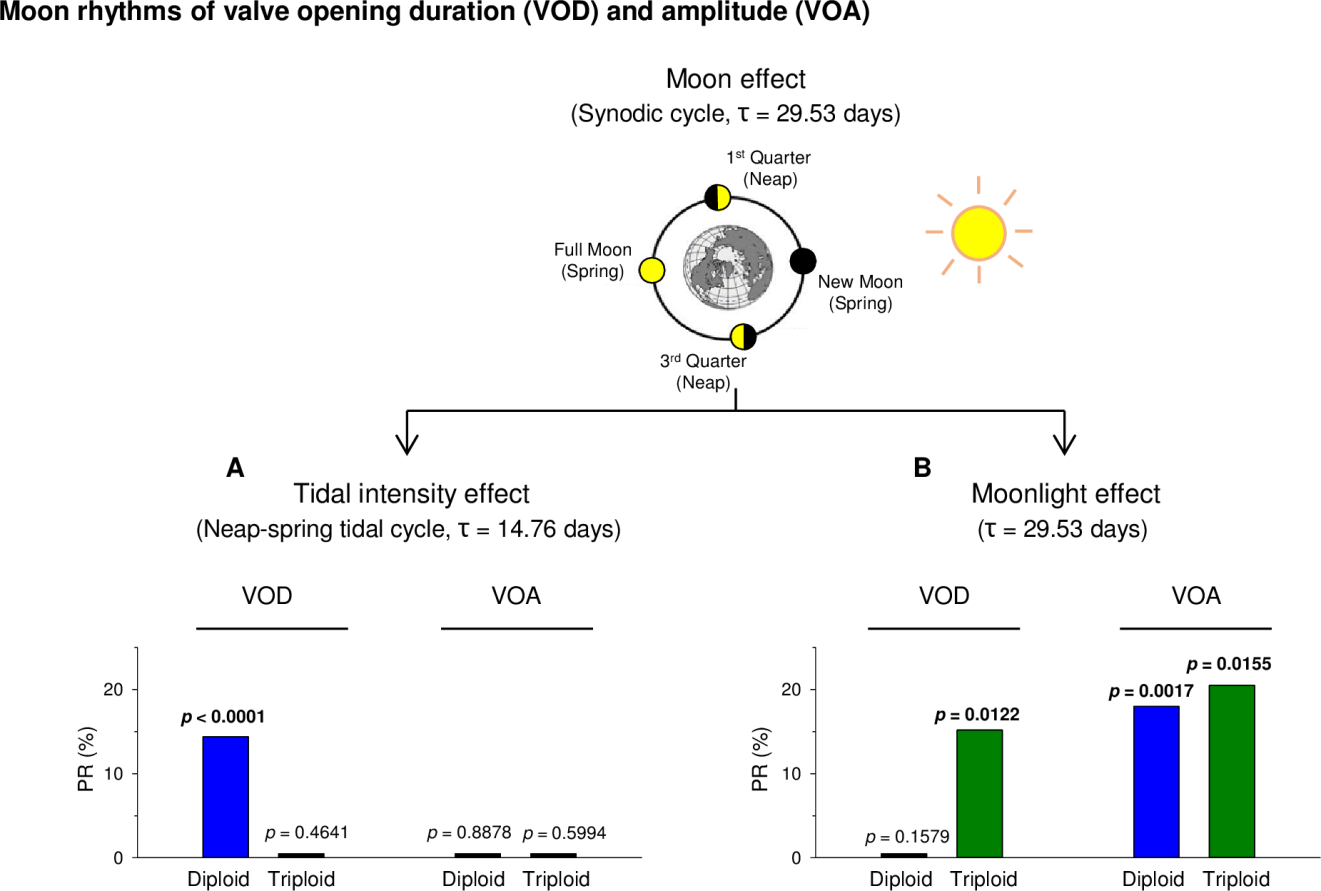
Circalunar rhythms. **(**A) Percent rhythms (PR, %) of neap-spring tidal cycle (tidal intensity effect, τ = 14.46 d)) during the 1-year analysis of VOD and VOA in diploid (n = 8, blue) and triploid (n = 8, green) oysters. (B) Percent rhythms (PR, %) of the entire synodic cycle (moonlight cycle, τ = 29.53 d) during the 1-year analysis of VOD and VOA in diploid (blue) and triploid (green) oysters. *P*-values indicate significance of the Cosinor model. Zero percent rhythms correspond to an absence of significant rhythmicity.

Synodic lunar cycles reflect the duration of time required for the moon to align with the sun and the earth in the same order (τ = 29.53 days) (Fig 5). Due to this phenomenon, a moonlight cycle appears every lunar cycle, with a maximum of moonlight during the full moon and a minimum during the new moon. Moreover, the tidal intensity variation corresponds to neap-spring cycles, which occur two times/synodic month (τ = 14.76 days). The strongest tides (spring tides) occur during syzygy (full or new moon), whereas the lowest tides (neap tides) occur at first- and third-quarter phases of the moon. Chronobiological analyses on 1-year VOD indicated a significant neap-spring tidal cycle rhythmicity in diploid oysters but not in triploids (Fig 5A); and inversely, a significant moonlight effect in triploid oysters but not in diploids (Fig 5B). Then, chronobiological analyses on 1-year VOA showed no significant neap-spring tidal cycle rhythmicity for VOA in either diploids or triploids (Fig 5A). On the contrary, a moonlight effect appeared significant in both ploidies (Fig 5B).

### Spawning event

**Fig 6:**
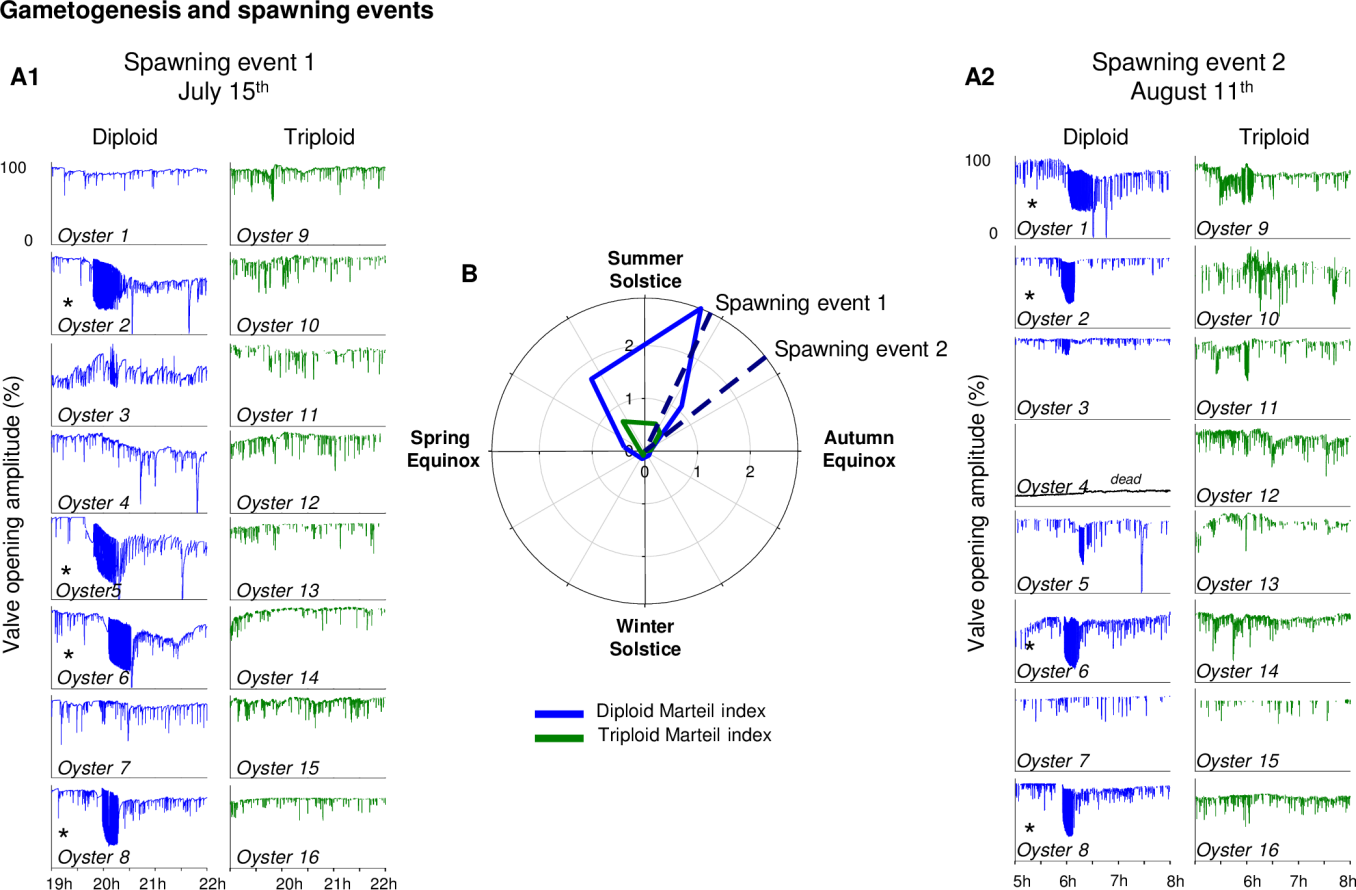
Gametogenesis and spawning events. **(**A) *In situ* spawning events recording during the 1-year analysis of diploid (blue) and triploid (green) oysters. The first spawning event was recorded in July (A1), the second in August (A2). Stars (*) indicate spawning events, *p*-value = 0.05. (B) Mean Marteil index measured on a dedicated group of diploid (blue) and triploid (green) oysters presented on a polar plot (7 measurements, n = 10 per time and ploidy).

All female spawning events characterized by valvometry were in summer (*i*.*e*. long days, decreasing daylength) (Fig 6). The 15^th^ of July, 2 days after the full moon, four diploid oysters (oysters 2, 5, 6 and 8) spawned in a synchronized manner, around 20 h (UTC), while no spawning events were observed in triploid oysters (Fig 6A1). The 11^th^ of August, 1 day after the full moon, four diploid oysters (oysters 1, 2, 6 and 8) spawned, again in a synchronized manner, around 6 h (UTC), while no spawning events were observed in triploid oysters (Fig 6A2). Thus, diploid females 2, 6 and 8 spawned twice in the same summer, while oyster 5 spawned only in July, and oyster 1 only in August. Mean gametogenesis levels were estimated with the Marteil index and showed increased gametogenesis throughout the spring, reaching a maximum of index 3 in summer just before the first spawning event (Fig 6B). In triploid oysters, the mean Marteil index revealed delayed and weak gametogenesis compared to diploids, never reaching index 1. In all oysters, the Marteil index was 0 at the beginning of autumn.

### Growth

**Fig 7:**
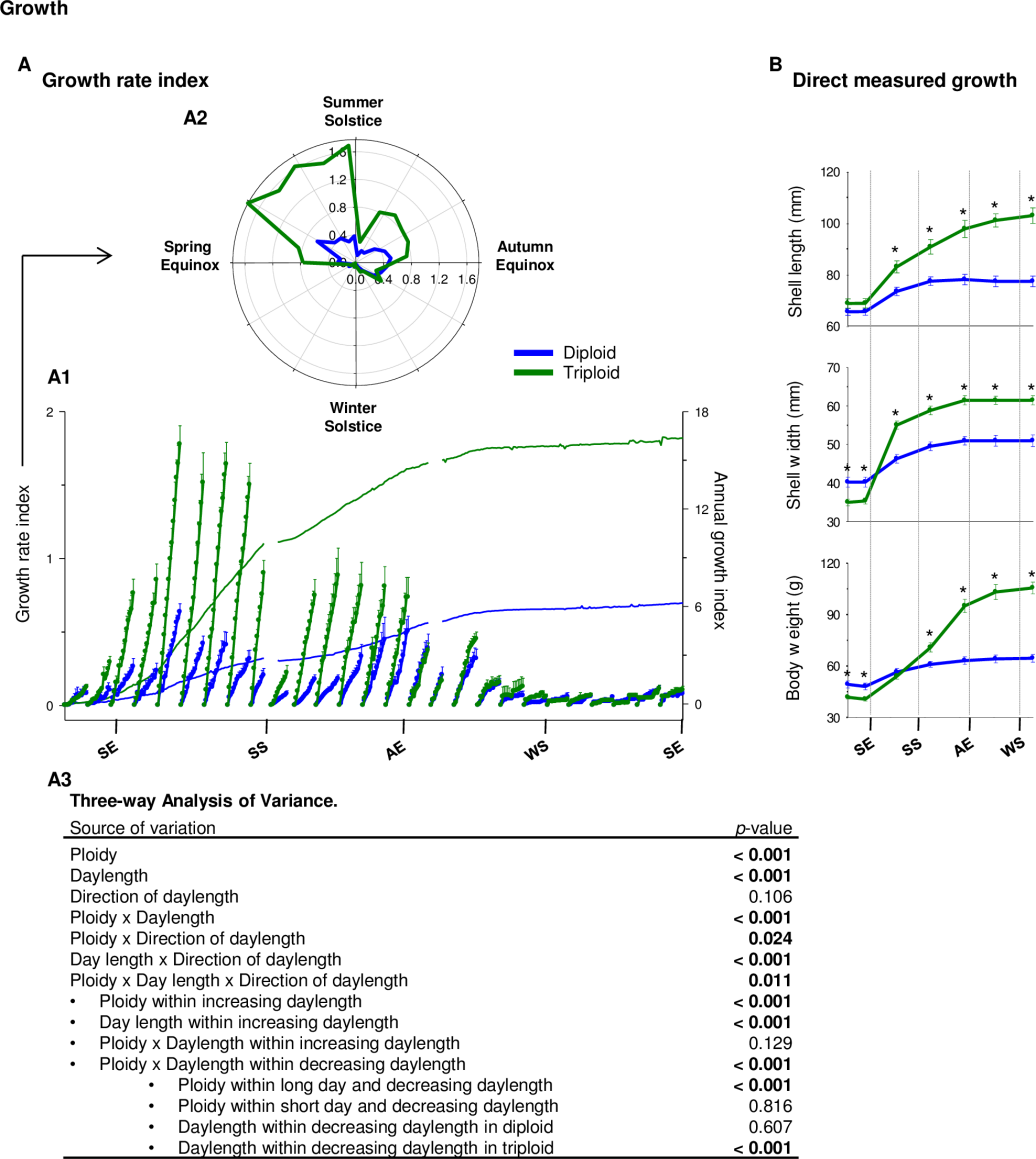
Growth. **(**A) Mean growth rate index in diploid (blue, n = 8) and triploid (green, n = 8) oysters, measured with valvometry HFNI. (A1) One-year (right axis) and 15-day (left axis) growth rate index in diploid and triploïd oysters. The x-axis shows time, with SE for summer equinox, SS for summer solstice, AE for autumn equinox, WS for winter solstice. (A2) Fifteen-day growth rate indices presented on a polar plot. (A3) Three-way analysis of variance on 15-day growth rate index using ploidy (diploid or triploïd), daylength (long or short days) and direction of daylength (increasing or decreasing daylength). (B) Direct measured growth on a dedicated group of oysters (7 measurements, n = 16 per ploidy). Stars (*) indicate significant differences between diploid and triploid oysters at a given time point. *P*-value = 0.05.

Fig 7A presents growth data collected with HFNI valvometry. Fig 7A1 shows the annual mean growth index (curves in background) of diploid and triploid oysters. In triploids, the index was ≈ 2.6 times higher than in diploids. Growth rate indexes based on the 15-day periods are presented in the same graph (Fig 7A1) and in a polar plot (Fig 7A2). At the annual scale, three-way ANOVA showed a significant effect of ploidy, a daylength effect (growth rate index higher during long days than during short days), but no direction of daylength effect on oyster growth rate index. There was also a significant interaction among the three factors. The ploidy effect was significant during increasing daylength (winter and spring), with a 3.6 times higher growth rate index in triploids in spring, when the index was maximum for both ploidies. During decreasing days, a significant ploidy effect was observed solely during long days, with a 2.8 times higher growth rate index in triploids in summer. There was no significant effect of ploidy in autumn. The daylength effect on growth rate index was significant only during increasing daylength, with a maximum in spring in both diploids and triploids. Direct measures (Fig 7B) on individual diploid and triploid oysters showed significant differences in morphogenesis traits. The change of the slope of the growth rate was in accordance with the valvometric growth index (Fig 7A1) and the direct measures (Fig 7B): maximum growth rate between the spring equinox and the summer solstice; intermediate growth rate between summer solstice and autumn equinox; very low growth rate between the autumn and spring equinoxes. Finally, in accordance with the growth rate index measured, the measures of shell length, shell width, and soft body weight were respectively 2.9, 2.5 and 4.2 times higher in triploid oysters after one year (Fig 7B).

## Discussion

The aim of this study was to investigate the relationship between the annual course of the photoperiod and seasonal behavior in diploid and triploid oysters. Animals react to annual changes in daylength and direction of daylength in order to anticipate environmental changes, using photoperiod to determine the timing of key physiological processes such as gametogenesis and spawning, growth and energy storage. In any given location, photoperiod is a much more reliable seasonal cue than temperature, food availability or rainfall, which vary greatly over time [9,48]. The underlying mechanisms of use of the photoperiod signal are still debated. Two major theories have been advanced, i) the oscillator model using the circadian clock, proposed by E. Bunning in 1936 [49] and ii) the hourglass model functioning without any internal clock, proposed by T. Lees in 1950 [50]. Recent studies in insects suggest that both mechanisms, not mutually exclusive, explain the measure of time at the annual scale [51].

In the present work, we study how ploidy (*i*.*e*. the number of copies of chromosomes) may introduce a difference in physiological and behavioral activities of the oyster *C*. *gigas*. It is well-known that most physiological processes at the annual scale in diploid (2 copies of chromosomes) *C*. *gigas* are organized as a function of the annual gametogenesis cycle; starting in the beginning of winter and finishing in summer with spawning events [11–14]. On the contrary, triploid oysters (3 copies of chromosomes) have been selected for commercial needs to avoid or reduce gametogenesis activity and favor growth [23–26].

### Gametogenesis and growth

A characteristic behavioral signature coupled with the Marteil index showed female spawning events and gametogenesis in diploid oysters. Moreover, we showed for the first time *in situ* to our knowledge that in a given individual, a spawning event could happen twice in the same summer. On the contrary, there was an absence of spawning events and much reduced gametogenesis in triploids, yet their annual shell growth rate was 2.6 (valvometric index) and 2.9 (direct measures) times higher than diploids.

The annual reproductive cycle of *C*. *gigas* seen here followed previous reports [11–14]. In diploid oysters, spring was characterized by active gametogenesis and summer by gonad maturation and spawning events. The lower, but not null, gametogenesis index of triploid oysters in spring and summer also agrees with previous studies: triploid *C*. *gigas* are generally considered sterile, yet partial gametogenesis events are sometimes recorded [25–27,52,53]; here the partial gametogenesis seen in triploids in spring and summer did not lead to spawning events. The initiation of gametogenesis was at the end of autumn / beginning of winter, and as the resorption period is in autumn (degenerating stage), this stage of gonadal proliferation could not be observed macroscopically [13]. This first stage of gonadal mitosis can differ between diploid and triploid oysters [25], and is sometimes delayed in triploids [52]. This winter initiation of gametogenesis appears of fundamental importance for a later benefit from the warm spring and summer temperatures indispensable for gonad development, maturation and spawning [11,53,54]. While it was demonstrated that gametogenesis cycles are shaped by photoperiod and/or temperature [13], at a sufficient temperature, spawning in *C*. *gigas* primarily occurs according to seasonal (summer), daily (morning or evening), circalunar and tidal (essentially at high tide of perigean spring tides) cycles [35]. Such synchronization has ecological consequences associated with gamete encounters and the dispersal of fertilized eggs, and this interplay of the many rhythms in a complex environment are in accordance with other results and widely observed in other marine organisms [55].

Maximum growth rate for all oysters was reached in spring, when days were long and increasing, and the maximal difference in growth rate between diploid and triploid oysters was highest in spring (3.6 higher in triploid). Growth rate was still important but slowed down in summer. Interestingly, decreased growth rate at the beginning of summer coincided in both diploid and triploid oysters with the first spawning event, although no spawns were observed in triploids. A hypothesis could be advanced that triploids and diploids use the same environmental signal (*i*.*e*. a photoperiod milestone, such as the summer solstice) to turn on physiological systems related to shell growth. Triploids thus have the same ability to respond to photoperiod changes, although a loss of spawning necessity leads to curtailed physiological action with respect to gametogenesis. Finally, these results confirm different energy allocations in diploids and triploids for different physiological needs.

### Photoperiod influences in both diploid and triploid oysters

First, independently of ploidy, photoperiod (daylength and direction of daylength) affect duration and amplitude of valve opening. Valve opening activity of many species is closely related to physiological processes such as respiration, nutrition, reproduction and responses to environmental stimuli [1]. In this study, results strongly suggest that photoperiod regulates *in situ* valve opening duration (VOD) and amplitude (VOA) of *C*. *gigas*, which clearly exhibited seasonality. This is opposite to the bivalve *Chlamys islandica*, an arctic scallop in which mean VOA and VOD values didn’t change throughout the year, despite very drastic seasonal changes [56]. In the present study, VOA and VOD for similar water temperatures were maximum in spring when day durations were long and increasing, and minimum in autumn when day durations were short and decreasing. In spring, increased VOA and VOD could be correlated to nutritional needs as food intake increases for growth and gametogenesis. In fact, a 20% increase of protein content in muscle was observed in diploid *C*. *gigas* in spring [57] and lipid content in gonads multiplied by three between the start of spring and summer spawning events [56]. As filter-feeders, oysters consume unicellular phytoplankton, which exhibit a seasonal cycle in temperate regions. After the winter period of lowest plankton concentration, spring is generally characterized by the highest abundance and by the most important algal blooms [58,59]. These supports the maximal growth rate in both diploids and triploids and increasing gonad maturation in diploids, occurring during spring. The seasonal variations in oyster valve behavior shown here suggest that photoperiodic signals allow an anticipation of seasonal changes in the food supply. Finally, the results also show that photoperiod influences seasonal modulations of rhythmic behaviors in both diploid and triploid oysters. As previously shown for VOD in diploids [3], here tidal cycle was the main driver of valve opening behavior, but for VOD and VOA and in both diploid and triploid oysters. However, during short days (winter and autumn) the tidal component of valve behavior was significantly stronger than during long days (spring and summer), revealing that photoperiod could modify the strength of the tidal rhythm in oysters. Autumn remained the season with the strongest tidal rhythm for both VOA and VOD behavior.

### Behavioral differences to photoperiod changes between diploid and triploid oysters

At the annual scale and independent of photoperiod, triploid amplitude of valve opening was lower than diploid. However, photoperiod differentially affected valve activity. During the long days of spring and summer, triploid VOD and daily rhythm were significantly higher than this of diploids. On the contrary, tidal rhythm was stronger in diploids. These significant differences disappeared during the short days of autumn and winter.

A dualism of daily rhythm had already been observed in *C*. *gigas*, switching from a nocturnal pattern during short days to a diurnal pattern during long days [4]. Here, we showed that in diploid oysters, the intensity of daily rhythm was also modulated by daylength (only for VOD) and by the direction of daylength (for both VOA and VOD). Interesting nuances were observed in rhythmic patterns between amplitude and duration of valve opening. Particularly, daily light / dark alternation was an important driver of diploid VOD in winter, but not on diploid VOA. In spring, daily rhythm was a weak driver for both VOD and VOA activity in diploids, while in triploids daily rhythm for VOA did not decrease in spring. Tidal rhythm of diploid behavior was modified by both daylength (stronger during short days) and direction of daylength (stronger during decreasing daylength), while tidal rhythm of triploids VOA was only modified by daylength. Rhythm is entrained by changes in daylength in many species, but the direction of daylength is also known as an entraining factor in other invertebrates including sea urchins [60] and fan mussels *Pinna nobilis* [61].

### Difference between triploids and diploids in response to the lunar synodic cycle

The synodic lunar month cycle is involved in two circalunar rhythms in marine organisms: the neap-spring tidal cycle rhythm and the moonlight rhythm. Here, a different impact was shown depending on ploidy and valve parameter. It was previously reported that the neap-spring tidal cycle, the two times per synodic cycle when the moon, sun and earth are in syzygy, i.e. alignment, during the new or full moon, significantly drives diploid VOD, increasing when tide coefficient increases, and vice versa [3]. In the present study, there was no significant effect of neap-spring tidal cycle on triploid VOD, meaning triploid opening duration was driven by tidal cycles but not modulated by their intensity. In the same way, no neap-spring tidal rhythm was observed in the amplitude of valve opening (VOA) in either diploids or triploids.

The synodic lunar cycle induces a moonlight cycle, with light intensity maximal at the full moon and minimal at the new moon. This cycle is well-known to drive moonlight cycles in marine organisms [62] such as corals [63,64], fishes [65], annelids [66], crustaceans and mollusks [1,67,68]. The moonlight rhythm has been reported to drive valve behavior in a marine bivalve, *Pinna nobilis* [1] but was observed here for the first time in the oyster *C*. *gigas*. Both diploids and triploids showed a moonlight rhythm of valve opening amplitude while only triploids exhibited a rhythm with valve opening duration.

Taken together, these results suggest that triploid oysters are more sensitive to light cues (moonlight rhythms and stronger daily rhythms in spring and summer) and less sensitive to tidal cues (no significant neap-spring tidal rhythm and weaker tidal rhythm in spring and summer) than diploids.

## Conclusion

Seasonal variations were observed in level of activity and rhythmic patterns of *C*. *gigas* valve behavior, as well as in growth and gametogenesis. Variations based on annual photoperiod and lunar rhythms exhibited significant differences according to ploidy. The molecular mechanism behind these differences remains to elucidate, but differences in gametogenesis and growth between diploid and triploid oysters is likely to be at least a part of the explanation. In future outlook, further studies using different triploid and diploid population origins would be useful to reinforce our conclusions about the difference of behavior according to a ploidy effect. The annual course of the photoperiod seems to act as an essential environmental cue synchronizing circannual/seasonal rhythms, however in mollusk bivalves, little is known about the potential relationship between a putative circannual clock and the outside world, which remains to study. These results suggest a complex interaction of tidal and daily rhythms with photoperiod, allowing optimization of seasonal biological processes through a selected sensitivity based on ploidy.
